# Beyond CTLA-4 and PD-1, the Generation Z of Negative Checkpoint Regulators

**DOI:** 10.3389/fimmu.2015.00418

**Published:** 2015-08-21

**Authors:** Isabelle Le Mercier, J. Louise Lines, Randolph J. Noelle

**Affiliations:** ^1^Department of Microbiology and Immunology, Geisel School of Medicine at Dartmouth, Lebanon, NH, USA

**Keywords:** cancer immunotherapy, autoimmunity, negative checkpoint regulators, TIM-3, LAG-3, TIGIT, BTLA, VISTA

## Abstract

In the last two years, clinical trials with blocking antibodies to the negative checkpoint regulators CTLA-4 and PD-1 have rekindled the hope for cancer immunotherapy. Multiple negative checkpoint regulators protect the host against autoimmune reactions but also restrict the ability of T cells to effectively attack tumors. Releasing these brakes has emerged as an exciting strategy for cancer treatment. Conversely, these pathways can be manipulated to achieve durable tolerance for treatment of autoimmune diseases and transplantation. In the future, treatment may involve combination therapy to target multiple cell types and stages of the adaptive immune responses. In this review, we describe the current knowledge on the recently discovered negative checkpoint regulators, future targets for immunotherapy.

## Introduction

T cells are initially stimulated through the T cell receptor (TCR) by the recognition of their cognate antigen presented by major histocompatibility complex (MHC) molecules. Optimal T cell activation requires a “second signal” provided by cosignaling molecules. Many of these molecules are members of the B7 family, and they act as rheostats that control the threshold for whether a given TCR interaction leads to activation and/or anergy. Positive costimulatory signals promote T cell proliferation and acquisition of effector function. CD28 is one such molecule that facilitates fulminant T-cell activation upon recognition of its ligands CD80 and CD86 at the surface of mature antigen-presenting cell (APC). Negative checkpoint regulators (NCRs) are molecules that down-regulate immune responses to prevent out-of-proportion immune activation, minimize collateral damage, and maintain peripheral self-tolerance.

The two NCRs that have been most actively studied are cytotoxic T lymphocyte (CTL)-associated antigen 4 (CTLA-4, CD152) and programed cell death protein 1 (PD-1, CD279) (1). They regulate immune responses at very different levels and by very different mechanisms. CTLA-4 primarily regulates the amplitude of the early stages of T cell activation by both outcompeting CD28 in binding CD80 and CD86, as well as actively delivering inhibitory signals to the T cell. PD-1 predominantly regulates effector T cell activity within tissue and tumors where the immune response is ongoing. The FDA approval of the CTLA-4 blocking antibody, Ipilimumab (Bristol-Myers Squibb) in 2011 for the treatment of advanced melanoma, followed in September and December 2014 by the approval of two PD-1 blocking antibodies, Pembrolizumab (Merck) and Nivolumab (Bristol-Myers Squibb) mark the beginning of a new era for cancer immunotherapy.

Multiple additional NCRs have been discovered in the recent years. The complex nature of the NCR pathways is only now being appreciated. These represent new promising targets for therapeutic manipulation.

## TIM-3

The T-cell immunoglobulin and mucin-containing protein 3 (TIM-3) was initially identified as a specific marker of fully differentiated IFN-γ producing CD4 T helper 1 (Th1) and CD8 cytotoxic (Tc1) cells (2). TIM-3 expression is regulated by T-bet, a Th1 transcription factor (3). In addition to T cells, TIM-3 is also highly expressed on regulatory T cells (Tregs), monocytes, macrophages, and dendritic cells (DCs). TIM-3 shares a common structure with the other TIM family members consisting of an N-terminal IgV domain followed by a mucin domain, a transmembrane domain, and a cytoplasmic tail. TIM-3 does not contain any known inhibitory signaling motifs but its phosphorylation on two intracellular tyrosine residues allows for the recruitment of the Src family tyrosine kinase Fyn and the p85 phosphatidylinositol 3-kinase (PI3K) adaptor (4).

The S-type lectin Galectin-9 (Gal-9) has been identified as one of TIM-3 ligands, binding the IgV domain of TIM-3 (5). Gal-9 is a widely expressed soluble molecule upregulated by IFN-γ (6).

### TIM-3 negatively regulates Th1 and Tc1 responses

Binding of Gal-9 to TIM-3 causes an inhibitory signal, resulting in apoptosis of Th1 cells and cytotoxic CD8 T cells *in vitro* (5, 7).

TIM-3/Gal-9 blockade generally induces hyperproliferation of effector cells associated with increased Th1 cytokine production (5) and increased CD8 T cell cytotoxicity (8). As a consequence, blocking TIM-3-mediated signaling on T cells *in vivo* accelerates or exacerbates Th1- and Tc1-mediated diseases. Gal-9 siRNA-treated mice (5) and Gal-9 deficient hosts (9) present increased symptoms of experimental autoimmune encephalomyelitis (EAE), the mouse model of multiple sclerosis. In addition, both TIM-3 blocking antibody and TIM-3–Ig fusion protein exacerbate symptoms of EAE (5, 10, 11), type I diabetes in non-obese (NOD) mice (12), and acute graft-versus-host disease (aGVHD) (13, 14). Importantly, TIM-3 deficiency on donor T cells exacerbates EAE and aGVHD (10, 14). On the other hand, blocking this pathway can dampen allergen-induced airway inflammation by skewing the Th2 response toward a Th1 type (15).

Conversely, activating the TIM-3 pathway ameliorates various disease models. Gal-9 overexpressing mice are protected from aGVHD (14). Recombinant Gal-9 administration suppresses EAE (5, 9) and prolongs the survival of fully allogeneic skin or cardiac transplants (16–18). Gal-9 expressing islets are also protected from rejection by NOD T cells (19). In all these models, the protection conferred by Gal-9 is associated with a decrease in IFN-γ producing Th1 and/or Tc1 cells.

Taken together, these data strongly support the hypothesis that the upregulation of TIM-3 on activated T cells and its interaction with Gal-9 plays a critical role in attenuating and/or terminating both CD4 Th1 and CD8 Tc1 immune responses.

### TIM-3 regulates Th17/Tregs differentiation

Whether and how TIM-3 and Gal-9 regulate Th17 cells is unresolved. While some studies show a negative effect of Gal-9 on both Th1 and Th17 development *in vivo* (16, 20), some studies show an impact on Th1 only (19). Gal-9 potentiates Treg conversion, and suppresses differentiation of Th17 cells *in vitro* (20, 21). As a result, Gal-9 administration ameliorates collagen-induced arthritis (CIA) by decreasing the levels of IFN-γ and IL-17 in the joints (20). However, one study demonstrated that Gal-9 suppression of Th17 development is TIM-3-independent (9).

*In vitro*, blocking TIM-3 promotes both Th1 and Th17 cytokine production by human and mouse CD4 T cells (8, 22). Similarly, *in vivo* TIM-3 blockade increases both Th1 and Th17 cells (8). However, TIM-3 blockade does not increase incidence and severity of Th17-mediated EAE but alters the pattern of inflammation due to differential effects on Th1 versus Th17 cells (10). TIM-3 blockade also inhibits Treg differentiation *in vitro* (8) and *in vivo* (12). As a result, TIM-3 deficient mice cannot be tolerized by high-dose aqueous antigen administration (11) and TIM-3 blockade abrogates Treg-mediated tolerance to allogeneic islets induced by donor-specific transfusion and costimulatory blockade (12).

Overall, evidence suggests that TIM-3 and Gal-9, possibly independently of each other, are involved in the differential regulation of Tregs and Th17 differentiation and contribute to T cell tolerance. One mechanism proposed is that TIM-3 negatively regulates IL-6 production by CD4 T cells. Therefore, blocking TIM-3 induces IL-6 production, which then antagonizes Treg differentiation and promotes IL-17 production by naive CD4 T cells (8).

### TIM-3 regulates innate cell activation/expansion

TIM-3 is highly expressed by innate immune cells including monocytes, macrophages, and DCs, and regulates their function in several ways.

In some circumstances, TIM-3 acts as a negative regulator of myeloid cell activation. Monney et al. first showed that a blocking TIM-3 antibody induces increased activation of macrophages (2). In addition, TIM-3 blockade during the innate immune phase of the response to coxsackievirus B3 (CVB3) infection exacerbates inflammatory heart diseases (23).

TIM-3 expression on macrophages can dampen TLR4-mediated inflammatory reactions and damage (24). Moreover, expression of TIM-3 and TLR4 is reciprocally regulated (25, 26). TIM-3 blockade enhances macrophage responsiveness to LPS stimulation, exacerbates sepsis (24), and enhances ischemia reperfusion injury damage in mouse liver transplantation (27). In these cases, the effect of TIM-3 blockade is dependent on intact TLR4 expression. TIM-3 overexpression on macrophages as observed in chronic hepatitis C virus (HCV) infection, or by transgenic overexpression, is associated with diminished cytokine production upon stimulation (24, 26). However, TIM-3 overexpressing macrophages in hepatocellular carcinoma patients promote tumor cell growth via IL-6 production (28).

On the other hand, several studies have indicated that TIM-3 can promote activation and inflammatory cytokine production by innate immune cells. Triggering the TIM-3 pathway on DCs and monocytes via Gal-9 treatment or agonistic anti-TIM-3 antibody synergizes with TLR ligands to promote their activation (29). Gal-9 alone promotes the secretion of proinflammatory cytokines by TIM-3 expressing human and mouse monocytes and DC (30). In addition, Gal-9 treatment reverses immune suppression in tumor-bearing hosts and enhances survival by promoting the maturation of TIM-3 expressing DCs thus promoting adaptive immunity (31). These apparently contradictory results might be explained by a differential effect of TIM-3 on macrophages versus DCs. Other binding partners for TIM-3 and/or Gal-9 can also mediate this differential effect. In addition, the fact that TIM-3 and Gal-9 can act as both receptors and ligands with regard to signaling has to be taken into account.

Several reports have indeed established that there is a reciprocal signal transmitted to the Gal-9 expressing innate immune cell. Therefore, a TIM-3–Ig fusion protein often used as a blocking reagent may trigger a signal to Gal-9 expressing cells independently of its blocking of TIM-3. For example, the interaction of Gal-9 expressed on macrophages with TIM-3 expressed on Th1 enhances their bactericidal activity. This effect can be mimicked on macrophages by treatment with TIM-3–Ig (32, 33). Importantly, this effect is lost in Gal-9-deficient macrophages (32). Kuchroo et al. created TIM-3 Tg mice where TIM-3 overexpression is controlled by the human CD2 promoter and restricted to T cells. These mice display dampened T cell immunity resulting in increased tumor progression that is linked to the expansion of granulocytic myeloid-derived suppressor cells (MDSCs). Thus, TIM-3 expressed on T cells is sufficient to trigger a signal via Gal-9 in MDSCs and promote their expansion (34). In conclusion, both TIM-3 and Gal-9 are expressed and can signal in innate immune cells. Thus, cis and trans interactions might occur and the final effect results from the integration of all of these signals (35).

### Additional ligands and partners for TIM-3 and Gal-9

As mentioned above, TIM-3 and Gal-9 may each have multiple binding partners. Several studies have found that Gal-9 may act through counter structures that are not TIM-3. First, Gal-9-mediated cell death of Th1 cells is not completely abolished in TIM-3-deficient cells (5). In addition, Gal-9 induces proinflammatory cytokine production by T helper cells, suppresses Th17 development, and induces plasma cell apoptosis in a TIM-3-independent manner (9, 36, 37). A recent study also reported that TIM-3 does not act as a binding partner for Gal-9 on human T cells (38).

Early crystal structure studies of TIM-3 have revealed a Gal-9 independent ligand-binding surface in the IgV domain (39) and several TIM-3 additional binding partners have since been uncovered. Like other members of the TIM family, TIM-3 also binds phosphatidylserine (PtdSer), exposed at the surface of apoptotic cells (40). Binding to PtdSer by TIM-3 mediates the uptake of apoptotic cells by TIM-3 expressing phagocytes such as CD8α DC and appears crucial for the clearance of apoptotic cells *in vivo* (41). TIM-3 blockade thus prevents uterine macrophages from clearing apoptotic cells and the resulting local inflammation increases fetal resorption (42).

TIM-3 also interacts with the high-mobility group protein B1 (HMGB1). This interaction prevents the trafficking of nucleic acids into endosomes and decreases stimulation of endosomal TLR pathways thereby preventing tumor recognition by TIM-3 expressing DCs and promoting tumor escape (43).

Finally, TIM-3 has recently been described to interact with the carcinoembryonic antigen cell adhesion molecule 1 (CEACAM-1) in both cis and trans through their N-terminal domains. CEACAM-1 endows TIM-3-mediated inhibitory function by facilitating TIM-3 surface expression (44). In conclusion, TIM-3 and Gal-9 can both use multiple binding partners mediating various outcomes in both T cells and innate immune cells unraveling a very complex functional role.

### TIM-3 regulates T cell exhaustion

TIM-3 expression has been described to mark the most dysfunctional CD8 T cells in various chronic viral infections in both human and mice such as human immunodeficiency virus (HIV), HCV, and lymphocytic choriomeningitis virus (LCMV) (45–47). This state of T cell dysfunction, called T cell exhaustion, caused by chronic antigenic stimulation is characterized by the failure to respond further, proliferate, and exert effector functions such as cytotoxicity and cytokine secretion in response to antigen stimulation. These cells as discussed thereafter, often co-express other inhibitory molecules.

Similarly, TIM-3 and PD-1 are co-expressed on most CD4 and CD8 T cells infiltrating solid tumors or in hematologic malignancy in mice and these cells are dysfunctional (48, 49). TIM-3 and PD-1 expression is also upregulated on exhausted tumor-specific CD8 T cells in the blood of melanoma and lymphoma patients (50, 51). In both chronic viral infection and cancer, blocking TIM-3 *ex vivo* or *in vivo* increases the functionality of exhausted T cells and synergizes with PD-1 blockade to restore viral control (46, 52) or to inhibit tumor growth (48, 49).

TIM-3 expression on tumor-infiltrating lymphocytes (TILs) also defines highly suppressive Tregs in both human and mouse tumors (53–55). As a result, TIM-3 blockade and Treg depletion have a synergistic effect on tumor growth inhibition (54).

## LAG-3

The lymphocyte-activated gene-3 (LAG-3, CD223) is a surface molecule highly homologous to CD4 in structure, but with less than 20% identity at the amino acid level (56). Like CD4, LAG-3 binds to MHC class II molecules, but with a much higher affinity (57).

LAG-3 is expressed on activated CD4 and CD8 T cells, and on activated Tregs (58) and Tr1 cells (59, 60). It is also expressed on a subset of NK cells (61) B cells (62) and plasmacytoid DCs (63). In resting T cells, LAG-3 is localized and degraded within the lysosomal compartments (64, 65). After stimulation, LAG-3 is rapidly translocated to the cell surface where its expression is regulated by two TCR-induced metalloproteases, ADAM10 and ADAM17. LAG-3 cleavage from the cell surface by these metalloproteases allows for normal T-cell activation (66). As a result, LAG-3 is only transiently expressed at the surface of activated T cells stimulated in acute conditions, although it remains high on T cells stimulated within tolerizing environments (58, 67).

LAG-3 associates with the TCR:CD3 complex following TCR engagement and negatively regulates signal transduction (68). A single lysine residue (K468) within a conserved “KIEELE” motif in the cytoplasmic tail of LAG-3 is essential for interaction with downstream signaling molecules and inhibitory function (69).

### LAG-3 prevents autoimmunity in mice

LAG-3 deficiency alone does not induce autoimmunity in non-autoimmune-prone mouse strains (70–72) and does not induce major alterations in T cell development or function but a reduced NK cell cytotoxicity (70). Probably for that reason, LAG-3-deficient mice present a reduced ability to control tumor growth (72). However, LAG-3 blockade or LAG-3 deficiency accelerates diabetes in the predisposed NOD mice (71, 73). In addition, combined LAG-3 and PD-1 deficiency induces massive autoimmune conditions and early death in several different genetic backgrounds (71, 72). These clearly identify LAG-3 as a non-redundant negative T cell regulator.

### LAG-3 negatively regulates T cell activation

Multiple evidences suggest that LAG-3 signaling directly inhibits primary activation of T cells *in vitro* and *in vivo*.

LAG-3 blockade induces increased proliferation and cytokine production by T cells activated *in vitro* (74, 75). *In vivo*, LAG-3-deficient T cells exhibit a delay in cell cycle arrest resulting in a larger memory T cell pool following simian virus (SV) infection (76). LAG-3-deficient donor T cells also induce more severe aGVHD due to increased proliferation and enhanced effector functions (77).

LAG-3 also plays both a direct role and an indirect role in maintaining the tolerogenic state of CD8 T cells *in vivo*. LAG-3 deficiency on CD8 T cells prevents the development of transgenic CD8 T cell tolerance, these cells are exposed to cognate self-Ag (78). In a different model of CD8 T cell tolerance induced by allogeneic bone marrow transplantation and costimulation blockade, LAG-3 blockade also abrogates donor-specific CD8 T cell tolerance (79). However, in this system, LAG-3 is not intrinsically required on CD8 but must be expressed by other cells.

### LAG-3 regulates the induction and suppressive ability of Tregs and Tr1 cells

LAG-3 is a marker of IL-10 producing Tr1 cells in both mice and humans (59, 60). Importantly, LAG-3 is one of the most overexpressed genes on CD4 transgenic T cells stimulated within a tolerizing environment (58, 78).

LAG-3 likely plays a crucial role in Tr1 induction and its function as ectopic LAG-3 expression in CD4 T cells confers a suppressive activity and blocking LAG-3 inhibits the suppressive function of Tr1 cells *in vitro* and *in vivo* (58). LAG-3 crosslinking on human T cells also induces a functional unresponsiveness that can be reversed by IL-2, consistent with a Tr1 phenotype (80).

LAG-3 is also highly expressed by activated natural Tregs (58). LAG-3 plays a role in modulating Treg induction/expansion as LAG-3 deficiency on T cell or LAG-3 blockade prevents Treg conversion in favor of a TH1 skewing (81). The importance of LAG-3 for Treg-mediated suppression is controversial. In one study, LAG-3-deficient Tregs cannot suppress homeostatic proliferation (82), whereas two other studies showed no difference in LAG-3-deficient or -sufficient Tregs to suppress homeostatic proliferation and aGVHD (77, 81). It is possible that LAG-3 is necessary for Treg-mediated suppression at high Effector/Treg ratios while being dispensable at lower ratios.

In these last two studies, LAG-3 expression on conventional T cells however, regulated their susceptibility to Treg-mediated suppression. LAG-3-deficient T cells undergo increased homeostatic expansion when transferred in a lymphopenic host (82) and LAG-3 blockade also increases homeostatic expansion but only if Tregs are present (81). This involves a novel Treg-mediated suppression mechanism following MHC class II acquisition by Tregs through trogocytosis and subsequent inhibition of LAG-3 expressing conventional T cells (77). Thus, LAG-3 appears as a crucial molecule involved in both the development and function of suppressive T cells.

### LAG-3 regulates innate cell activation

Similar to other NCRs, LAG-3 is bidirectional in its signaling capacity and modulates DC activation by inducing downstream signaling via MHC class II molecules. LAG-3 expressed on activated T cells induces DC maturation with the production of TNFα and IL-12 *in vitro*. As a result, LAG-3 blockade in DC:T cell cocultures, prevents DC activation, and inhibits rather than increases T cell proliferation (83). This T-cell-mediated effect on DC can be mimicked by soluble LAG-3-Ig fusion protein (84). As a result, LAG-3–Ig acts as an adjuvant increasing Th1 and cytotoxic T cell responses to soluble antigen *in vivo* (85). Similarly, LAG-3–Ig administered together with irradiated tumor cells induces tumor regression and increases tumor cell-specific CD8 T cell responses (86). However, during Treg:DC interactions, LAG-3 engagement with MHC class II inhibits DC activation. In this case again, LAG-3-mediated signaling is not required but its binding to MHC II molecules initiates an inhibitory signaling pathway that suppress DC maturation (87). MHC II engagement through LAG-3 or crosslinking induces several pathways that have to be finely regulated to lead to cell activation or inhibition (88). Additional signals differentially expressed by activated T cells and Tregs such as CD40L might also influence the outcome of this interaction (83).

### Additional LAG-3 ligands

As for other NCRs, two other binding partners for LAG-3 have been described which are expressed in the tumor microenvironment: the Liver sinusoidal endothelial cell lectin (LSECtin) and Galectin-3 (Gal-3). Engagement of LAG-3 by LSECtin expressed in melanoma cells inhibits IFNγ production by effector T cells and increases IL-10 production by Tregs (89). Gal-3, a galactoside-binding soluble lectin is expressed in several cell types and involved in a broad range of physiological and pathological processes. Gal-3 binds to LAG-3, and LAG-3 expression is necessary for Gal-3-mediated suppression of tumor-specific CD8 T cells. Gal-3 deficiency on both T cells and the host improves tumor-specific CD8 T cell response suggesting both cis and trans interactions between the two molecules (90).

### LAG-3 regulates T cell exhaustion in cancer and chronic infections

In addition to PD-1 and TIM-3, LAG-3 is also upregulated and maintained in exhausted T cells in both chronic viral infections and cancer. LAG-3 is upregulated on virus-specific CD8 T cells in chronic LCMV infection. A functional role of LAG-3 in exhaustion is suggested by the fact that LAG-3 blockade synergized with PD-1 blockade to reverse exhaustion and improve viral control (91). LAG-3 expression on HIV-specific CD4 and CD8 T cells is also correlated with disease progression. Interestingly in HIV patients, LAG-3 and PD-1 are expressed on distinct subsets of exhausted T cells (92). LAG-3 and PD-1 are co-expressed on TILs in ovarian and on tumor-specific CD8 T cells in the blood of ovarian cancer patients (93). LAG-3 and PD-1 are also co-expressed on CD4 and CD8 TILs in various mouse tumor models (72). LAG-3 blockade alone does not always reverse the exhausted phenotype but can synergize with PD-1 blockade to improve effector functions and control viral load (91, 92) or induce tumor regression (72, 93).

## TIGIT

The T cell immunoreceptor with Ig and ITIM domains (TIGIT/Vstm3/WUCAM/VSIG9) is a novel member of the immunoglobulin super family (IgSF). TIGIT was recently identified by two independent groups through a genomic search for genes specifically expressed in T cells and bearing a structure similar to other immunomodulatory receptors (94, 95). TIGIT is a type 1 transmembrane protein containing an IgV extracellular domain and an immunoglobulin tail tyrosine (ITT)-like phosphorylation motif followed by an immunoreceptor tyrosine-based inhibitory motif (ITIM) in the cytoplasmic tail.

TIGIT pairs with CD226/DNAM-1 (DNAX Accessory Molecule-1) to form an emerging pathway that has striking similarities to the CTLA-4/CD28 pathway. CD226 and TIGIT bind the same set of ligands, the two nectin-family members poliovirus receptor (PVR) (CD155/Necl-5/Tage4) and poliovirus receptor-related 2 PVRL2 (CD112), and compete with each other (96). Both ligands are members of the nectin-like family, are widely expressed outside the hematopoietic system on fibroblasts and endothelial cells, and are involved in cell adhesion and motility. Notably, PVR is overexpressed in several tumor cells types (97, 98) and can be induced by Ras activation and genotoxic stress (99, 100). PVR is also induced by TLR ligand-activated APCs (101). Whereas CD226 is widely expressed on most immune cells (102), TIGIT is absent on naive T cells, but is expressed on activated and memory T cells, Tregs (94) and on NK cells and NKT cells (95) in mice and humans. Human TIGIT engagement by PVR induces a tyrosine phosphorylation on the ITT domain. This results in the recruitment of the phosphatase SHIP1 through different cytosolic adaptors leading to inhibition of phosphatidylinositol 3-kinase (PI3K), MAPK, and NF-kB signaling (103, 104). In mice, phosphorylation of either the ITT or the ITIM domain is sufficient for TIGIT-mediated inhibition (105).

### CD226 costimulates NK and T cell responses

PVR recognition by CD226 potentiates CD8 T cell and NK cell cytotoxicity toward tumor cells (97, 106, 107). Notably, CD226 is a crucial costimulatory molecule for CD8 T cells when activated by non-professional APC such as B cells but is dispensable when T cells are activated by professional APCs (108). As a result, CD226-deficient mice have impaired anti-tumor and antiviral T cell responses (109, 110). Upon engagement, CD226 is phosphorylated and interacts with LFA-1 inducing their recruitment to lipid rafts (111–114). CD226 deficiency thus impairs immunological synapse formation between CD8 T cells and target cells preventing the deliverance of the cytotoxic payload necessary for target cell killing (115). CD226 also regulates CD4 T cell expansion and cytokine production. CD226 blockade decreases Th1 differentiation and suppresses EAE while PVR deficiency decreases Th2 polarization (101, 102).

The polymorphism variant Gly306Ser of CD226 has been associated with susceptibility to multiple autoimmune diseases such as SLE, autoimmune thyroid disease, Type 1 diabetes, MS, and Celiac disease (116, 117).

### TIGIT has T-cell-intrinsic inhibitory function

Several reports attest that TIGIT negatively regulates T cell activation. While TIGIT deficiency alone does not induce overt autoimmunity, TIGIT pathway blockade exacerbates several immune diseases. TIGIT-deficient mice are more susceptible to EAE (118) and blocking TIGIT results in more rapid CIA and EAE diseases onset (96). TIGIT deficiency also induces neurological dysfunction in EAE susceptible, myelin oligodendrocyte glycoprotein (MOG)-specific TCR transgenic 2D2 mice (118). Finally, TIGIT-deficient T cells induce more severe GVHD (96).

Conversely, increased TIGIT function on T cells has been shown to ameliorate a variety of autoimmune disease models in mice. Soluble TIGIT decreases CIA and mice overexpressing TIGIT in T and B cells are protected against EAE (96). Lentiviral overexpression of TIGIT in CD4 T cells decreases their ability to mediate bone marrow damage and lengthens survival time in a mouse model of aplastic anemia (119).

The mechanism of TIGIT-mediated T cell inhibition is unclear. Some evidences suggest that TIGIT can directly inhibit T cell activation. *In vitro*, TIGIT engagement by an agonistic antibody decreases both human and mouse T cell activation when stimulated with anti-CD3 and anti-CD28 (119, 120). Conversely, TIGIT knockdown increases T cell proliferation and effector cytokine production while decreasing IL-10 production (119). This suggests that the negative downstream signaling via TIGIT could arrest T cell activation. However, TIGIT can also indirectly inhibit T cell activation by opposing the CD226-mediated positive costimulatory signal either through ligand competition or CD226 inhibition. As evidence, when T cells are activated with PVR transfected artificial APCs, a blocking TIGIT antibody increases T cell proliferation while soluble TIGIT-Ig decreases it (96). TIGIT deficiency or blockade also increases T cell proliferation to anti-CD3 and PVR-Ig stimulation. Importantly, when T cells are activated in the presence of PVR, TIGIT-mediated inhibition seems dependent on CD226, as CD226 blockade annihilates the positive impact of TIGIT blockade (121). FRET studies indicate that TIGIT and CD226 directly interact at the cell surface and that this interaction impairs CD226 homodimerization and function (121).

### TIGIT regulates DC immunostimulatory function

As mentioned before, most immunoregulatory molecules function in a bidirectional way and TIGIT also induces a reciprocal signal in PVR expressing APC. PVR engagement on DC with TIGIT-Ig induces IL-10 while suppressing pro-inflammatory cytokines production such as IL-12. Upon TIGIT ligation, PVR is phosphorylated and elicits downstream signaling in DCs, which then inhibits T cell responses by producing IL-10. In addition, TIGIT blockade exacerbates T cell responses only in the presence of DC. Finally, TIGIT–Ig-mediated inhibition of delayed type hypersensitivity (DTH) reactions *in vivo* is dependent of IL-10 (94). All these suggest that TIGIT negative regulation of T cell is indirect and mediated at least in part through modification of the immunostimulatory function of APCs.

In addition, TIGIT also exerts a direct inhibitory role on APCs as TIGIT-deficient APCs are better at promoting T cell proliferation. The maximal APC-induced T cell proliferation is achieved by combining both deficient T cells and deficient APCs suggesting that TIGIT has synergistic roles on T cells and APCs (118).

### TIGIT negatively regulates NK cell effector function

In addition to T cell inhibition, TIGIT negatively regulates NK cell cytotoxicity and cytokine production. TIGIT blockade increases NK cell cytotoxicity toward PVR expressing targets. Interestingly, TIGIT inhibition is dominant over the coactivation mediated by CD226 on NK cells (95, 105) whereas the net effect of PVR-T-cell interaction appears costimulatory (96). This might be due to additional inhibitory mechanisms preventing NK cytotoxicity. Indeed, NK cells also express CD96, another inhibitory receptor that also competes with CD226 for PVR binding (122). In addition, only co-blockade of TIGIT and MHC class I increases NK-mediated cytotoxicity against fibroblasts. TIGIT may thus represent an “alternative self” mechanism for MHC class I inhibition, preventing damage to self-tissue (95). Using mutants of TIGIT transfected into a YTS NK cell line and PVR transfected 7721.221 target cells, Fan et al. deciphered the mechanisms involved in TIGIT-mediated inhibition of NK cells. In these cells, direct TIGIT-mediated negative downstream signaling results in impaired granule polarization (104) and IFN-γ production (103).

### TIGIT promotes treg differentiation and defines activated, highly suppressive Tregs

TIGIT transcription is directly regulated by FoxP3 (123). TIGIT promotes inducible Treg differentiation as TIGIT deficiency decreases Treg conversion *in vitro*. Conversely, T cells overexpressing TIGIT generate greater frequencies of Tregs (124). *In vivo*, TIGIT expression defines a subset of activated natural Tregs with superior suppressive capacity in both humans and mice (125). TIGIT-expressing Tregs express higher amount of PD-1, CTLA-4, LAG-3, and TIM-3 and produce more IL-10 and Fibrinogen-like protein 2 (Fgl2). Notably, Fgl2 production is induced by TIGIT engagement and is responsible for the increased suppressive ability of TIGIT Tregs. These Tregs specifically suppress Th1 and Th17 responses *in vivo*, while promoting Th2 responses in an Fgl2-dependent manner (124). In human, co-expression of TIGIT and Fc receptor-like protein 3 (FCRL3) identifies Helios + memory Tregs (125, 126). Thus, as other NCRs, TIGIT is critically involved in Treg development and suppressive function.

### TIGIT regulates T cell exhaustion

Similar to PD-1, TIM-3, and LAG-3, TIGIT is upregulated on exhausted T cells in both chronic viral infections and cancer (121). A gene signature-based approach identified TIGIT expression as a marker for tumor-associated T cells. The TIGIT:CD3 ratio is increased on T cells in multiple human tumors compared to the corresponding normal tissues indicating that TIGIT is specifically upregulated in tumor-infiltrating T cells. Indeed, TIGIT is highly expressed on CD8 T cells co-expressing PD-1 infiltrating non-small cell lung carcinoma (NSCLC) and colorectal carcinoma (CRC) as well as several mouse tumor models. TIGIT is also elevated on CD4 and CD8 T cells in the blood of cancer patients. Whereas PD-L1 or TIGIT blockade alone have little effect, PD-L1 and TIGIT co-blockade dramatically improves CD8-mediated control of tumor growth leading to complete rejection in the majority of mice. Importantly only PD-L1 and TIGIT co-blockade elicit IFNγ and TNFα production by CD8 TILs (121).

In mice chronically infected with the Clone 13 strain of LCMV, TIGIT is highly expressed by PD-1^high^ exhausted T cells and TIGIT blockade acts synergically with PD-1 blockade to improve CD8 T cell effector function and viral control. TIGIT conditional knock down in T cells also improves antiviral T cell response and viral control. However, as mentioned above, TIGIT inhibition of T cells seems mediated by CD226 disruption as CD226 blockade annihilates the curative impact of TIGIT and PD-L1 co-blockade on both tumor growth and chronic infection (121). Interestingly, CD226 expression is downregulated on virus-specific CD8 T cells in both human HIV and mouse LCMV chronic infection reinforcing the proposition that the costimulatory pathway mediated by CD226 is disrupted in chronically exhausted T cells (127).

## BTLA

B and T lymphocyte attenuator (BTLA) was identified as another NCR in the IgSF structurally related to CTLA-4 and PD-1 (128, 129). BTLA expression is limited to lymphoid tissues, with highest expression on B cells, but also significant on both αβ and γδ T cells, mature DCs, and macrophages (128, 129). BTLA is expressed on naïve T cells, transiently upregulated upon TCR engagement, but is down regulated on fully activated T cells. However, in similarity with LAG-3, BTLA remains high on T cells rendered anergic *in vivo* (130). Its absence on fully activated T cells distinguishes it from other B7 family members like PD-1 and CTLA-4 (130, 131). Unique for a IgSF member protein, is the counter-receptor that BTLA binds. BTLA binds to the herpesvirus entry mediator (HVEM), which is a member of the tumor necrosis factor receptor superfamily (TNFRSF) (132, 133). HVEM is expressed on T cells, B cells, NK cells, DCs, and myeloid cells (133). HVEM was originally described as a receptor for HSV glycoprotein D, but interacts with multiple other ligands in addition to BTLA, including CD160 and LIGHT. CD160 is another negative receptor on T cells (134), while LIGHT is a costimulatory molecule. Crosstalk across protein families and the use of multiple binding partners by each protein are indicative of the widespread complexity of the system of negative checkpoint regulation. Like PD-1 and CTLA-4, BTLA contains two conserved ITIMs in its cytoplasmic tail (129). BTLA engagement is associated with phosphorylation at those two ITIMs motifs resulting in the association of the Src homology domain 2 (SH2)-containing protein tyrosine phosphatases SHP-1 and SHP-2 (128, 129, 135) with either the TCR or the BCR.

### BTLA negatively regulates T cell activation

*In vitro* studies demonstrate a direct negative activity of BTLA on T cell proliferation and cytokine production. BTLA-deficient T and B cells show enhanced proliferation in response to anti-CD3 and anti-IgM respectively (128, 129). Retroviral overexpression of BTLA in DO11.10 cells or agonist BTLA antibody suppresses anti-CD3-induced IL-2 production (129, 135). In addition, HVEM–Fc fusion protein inhibits T cell proliferation in response to multiple agonists (132). Interestingly, BTLA signaling induced by agonist antibody can act on T cell proliferation as far as 16 hours after TCR engagement, suggesting that the critical point of impact is during the later, more stable T cell–APC interactions (135).

### BTLA regulates peripheral tolerance

BTLA deficient mice gradually develop elevated anti-self antibodies, an increased number of activated CD4 T cells in the periphery and inflammatory cell infiltration of multiple organs. This can progress to development of a hepatitis-like disease and overall reduced survival (136). Consistent with this breakdown in peripheral tolerance, BTLA-deficient mice are resistant to the induction of T cell tolerance to an oral antigen or to high-dose antigen administration (137). In addition, BTLA-deficient ovalbumin-specific OT-I CD8 T cells cannot be tolerized by encounter with their cognate antigen *in vivo* and cause diabetes in RIP-mOVA recipient mice (137). BTLA-deficient mice also present increased susceptibility to EAE (129) and mice deficient in either BTLA or HVEM present heightened T cell and NKT cell responses to Con A and exhibit increased morbidity and mortality to Con A-mediated T cell-dependent autoimmune hepatitis (138, 139). Conversely, BTLA engagement leads to the induction of tolerance. An agonistic BTLA antibody prolongs heart allograft survival by suppressing alloreactive T cell responses and inducing IL-10-producing Tregs (140). In addition, a single dose of agonist BTLA antibody prevents the development of GVHD if given at the time of transplantation (141). In these models, BTLA engagement seems to favor the expansion of Tregs over T effector cells, adjusting the balance toward tolerance.

### BTLA regulates innate immunity

More recent studies have shown that BTLA is also able to regulate multiple lineages of cells within the innate immune system. BTLA-deficient mice on the RAG-deficient background are less susceptible to *Listeria Monocytogenes* infection (142). The authors reported that the loss of BTLA in the CD8α DC compartment prevents Listeria expansion within these cells. It is known that CD8α + DCs are necessary for Listeria expansion and dissemination within the host. In wild-type mice, BTLA normally suppresses Fas/FasL signaling in DCs to allow high levels of Listeria to grow and thereby induce potent protective CD4 and CD8 responses. In BTLA-deficient DCs, where Fas/FasL is enhanced, Listeria burden is reduced as is the adaptive immune response keeping it in check. Therefore, BTLA can exert direct immunoregulatory effects within the DC compartment.

Possessing both a rearranged specific TCR and rapid innate functions, γδ T cells are poised between the innate and adaptive arms of the immune response. BTLA appears to play a major role in both their homeostasis and function. The pool of γδ T cells is maintained through competition for survival signals from IL-7 or IL-15. In γδ T cells, IL-7 increases BTLA levels on the cell surface, which in turn, restricts their expansion and negatively regulates IL-17 and TNFα. BTLA-deficient mice thus exhibit enhanced disease in a γδ T cell-dependent model of dermatitis whereas an agonistic BTLA antibody reduces inflammation (143).

A particular role for BTLA has been described for Vγ9Vδ2 T cells. This subset is thought to have functions in tumor immune surveillance. In human Vγ9Vδ2 T cells, BTLA interaction with HVEM–Fc fusion protein negatively regulates Vγ9Vδ2 T-cell proliferation to both TCR-dependent and -independent activation. During TCR-mediated activation, BTLA clusters to the TCR and decreases phosphorylation of ZAP-70 and Erk1/2. BTLA blockade increases TCR signaling and restores the ability of human Vγ9Vδ2 T cells to react to HVEM expressing lymphoma cells (144). Although these suppressive activities show similarity to PD-1 and CTLA-4, the primarily naïve and central memory expression of BTLA distinguishes it from these immunoregulators, and gives BTLA its own niche.

In a transfer model of colitis, HVEM expressed on a radioresistant cell population interacting with BTLA was found to be critical in preventing inflammation (145). Surprisingly, BTLA expression on the donor T cells had a minor impact, and BTLA expression was more important in the recipient RAG-deficient mice. These data suggest that these molecules should be examined outside of the usual T cell–APC focus.

### BTLA as a ligand demonstrates T cell pro-survival function

A wealth of reports demonstrates a negative receptor function for BTLA. However, like many of the immunoregulatory molecules that can act as both receptors and ligands, BTLA also appears to induce a reciprocal positive pro-survival signal in HVEM-expressing T cells. Indeed, BTLA has been implicated in sustaining the survival of HVEM-expressing effector and memory T cells in various mouse models (145–147). Particularly, during vaccinia virus infection, HVEM expression on T cells and BTLA expression on the host are both necessary for the development of a protective response. In this model, BTLA expressed by APCs functions as a ligand that delivers positive signals in trans to HVEM expressing T cells (147).

An important point is that T cells express both HVEM and BTLA and several reports also demonstrate that BTLA expression by T cells is also critical for their survival. In a GVHD model, transfer of BTLA-deficient donor cells results in an impaired anti-host response due to a loss in donor T cell survival (148). Similarly, BTLA-deficient T cells do not induce increased colitis when transferred into RAG-deficient mice because of an impaired accumulation (145). A positive correlation has also been described between BTLA expression and the ability of human T cells to generate recall responses to the Mycobacterium tuberculosis antigen Ag85B (149). Sakoda et al. confirmed these findings and showed that expression of the extracellular domain of BTLA is sufficient to restore the survival of BTLA-deficient T cells during GVHD, further demonstrating the role of BTLA on T cells as a ligand in this model (150). BTLA-deficient T cell survival is also rescued with BTLA–Fc fusion protein (150, 151). Cellular BTLA and BTLA–Fc induce the recruitment of TNF receptor-associated factor 2 (TRAF2) to HVEM, promoting NF-κB activation and cell survival (151). Importantly, BTLA function as a ligand to induce pro-survival signal in GVHD is associated with its expression on donor T cells but not on recipient cells (150). A BTLA–HVEM interaction in cis on T cell could explain these findings. However, one report demonstrates that HVEM–BTLA cis interaction competitively inhibits HVEM activation by ligands expressed in the surrounding microenvironment rather suggesting a bystander T:T trans interaction involved in the BTLA–HVEM-mediated survival effect (152).

### BTLA negatively regulates tumor immunity

HVEM is expressed in 26 of 40 melanoma cell lines and moderately to strongly expressed on 75% of human melanoma metastases (153). BTLA and PD-1 are co-expressed on tumor-specific CD8 T cells in melanoma patients (153, 154) and these cells are dysfunctional (154). However, contrary to PD-1, BTLA upregulation seems to occur independently of the functional exhaustion driven by high antigen load (154). BTLA can be downregulated by vaccination with peptide and CpG oligodeoxynucleotides with a resulting loss of sensitivity to HVEM-mediated suppression of cytokine production (153). Importantly, BTLA blockade synergizes with PD-1 and TIM-3 blockade in enhancing proliferation and cytokine production by tumor-specific T cells *in vitro* indicating a non-redundant role for BTLA (154). Similarly, BTLA blockade combined with active immunization enhanced anti-tumor immunity (155) and can lead to regression of large adenocarcinomas in mice (156). However, as discussed above, the effect of BTLA may not be completely negative. BTLA expressing TILs appear more proliferative to IL-2. This may be because BTLA expression is higher on newer central memory type cells that are less likely to be exhausted. In addition, BTLA positive cells present reduced sensitivity to activation-induced cell death. These data indicate that BTLA may extend the life of TILs, but also maintain quiescence (157).

## VISTA

The V-domain Ig suppressor of T cell activation (VISTA) also known as PD-1 homolog (PD-1H) has recently been identified by our group and others as a novel NCR in the IgSF. VISTA is a type I transmembrane protein with a single IgV domain with sequence homology to the IgV domains of the members of CD28 and B7 families (158, 159). VISTA cytoplasmic tail domain contains two potential protein kinase C binding sites as well as proline residues that could function as docking sites, suggesting that VISTA could potentially function as both a receptor and a ligand. VISTA does not contain ITIM-like motifs. Modeling algorithms have suggested homology to either PD-1 (158) or PD-L1 (159). The counter structures interacting with VISTA have not been identified yet.

Unlike PD-L1, VISTA expression is restricted to the hematopoietic compartment. It is constitutively and highly expressed on CD11b myeloid cells such as neutrophils, monocytes, macrophages, and DCs, and expressed at lower levels on naïve CD4 and CD8 T cells and Tregs both in humans (160) and mice (158, 159).

### VISTA regulates peripheral tolerance

The negative regulatory function of VISTA is clearly demonstrated in deficient mice. VISTA-deficient mice demonstrate an age-related proinflammatory signature, with elevated serum cytokines, spontaneous T cell activation, and chronic multi-organ inflammation. Nonetheless, single VISTA deficiency as well as VISTA/PD-1 and VISTA/PD-L1 double deficiencies do not induce overt autoimmunity in the absence of other predisposing factors (161–163).

However, VISTA deficiency on the 2D2 transgenic EAE susceptible background dramatically increases disease incidence and severity with 60% of mice dying by 2–3 months of age (163). Combined deficiency of VISTA and PD-1 further increases disease penetrance to 90% (162). On a non-susceptible background, VISTA blockade also accelerates EAE onset and severity (159).

VISTA appears to function both as a negative receptor on T cells and as a ligand expressed on APCs interacting with an unknown receptor on T cells. As evidenced, in a passive transfer model of EAE, VISTA deficiency on both T cells and host contribute to the control of autoimmunity with the most aggressive disease obtained by transferring VISTA-deficient pathogenic T cells into VISTA-deficient hosts. VISTA expression on the host, however, appears to contribute more than that on T cells as VISTA-deficient hosts always present accelerated disease regardless of the status of the T cells transferred (163). *In vitro*, maximal antigen-specific proliferation is achieved when both APCs and T cells are deficient, again supporting that VISTA on both T cells and APCs contributes to the inhibition of T cell proliferation via specific pathways (161).

### VISTA as a ligand negatively regulates T cell activation

Several findings suggest that VISTA negatively regulates T cell responses by acting as a ligand that interacts with an unknown receptor on T cells. A soluble VISTA–Ig fusion protein inhibits human and mouse CD4 T cell proliferation and cytokine production *in vitro* by suppressing early TCR activation (159, 160). A single dose of VISTA–Ig fusion protein prevents the development of GVHD in mice if given at the time of transplantation (158). When expressed on APCs, VISTA decreases antigen-specific T cell proliferation. VISTA blockade thus increases proliferation when T cells are stimulated with VISTA expressing myeloid APCs. Moreover, ectopic expression of VISTA on tumor cells interferes with protective anti-tumor immunity and allows increased tumor growth in vaccinated hosts (159).

### VISTA as a receptor negatively regulates T cell activation

Several findings also suggest a direct negative role of VISTA as a receptor on T cells. VISTA-deficient T cells respond by increased antigen-specific proliferation and cytokine production when compared to WT T cells *in vitro* and *in vivo*. On the other hand, VISTA engagement by an agonistic antibody suppresses antigen-specific proliferation when T cells are activated by VISTA-deficient APCs (161). In this system, because the APCs are deficient, VISTA on T cells functions independently of APCs to suppress T cell responses.

As a receptor on T cells, VISTA seems critical for the regulation of allogeneic responses. While an agonistic anti-VISTA antibody potently suppresses GVHD in mice (158), it does not prevent GVHD induced by VISTA-deficient donor T cells (164). In addition, VISTA-deficient T cells induce exacerbated GVHD with increased donor T cell expansion and decreased survival. However, VISTA-deficient recipient do not present aggravated GVHD, indicating that VISTA expression on recipient cells (APC) had little effect on the regulation of allogeneic T cells in this disease model (164).

### VISTA regulates treg differentiation and suppressive function

VISTA is also highly expressed by a subset of Tregs and like most NCRs seems to contribute to their differentiation and suppressive function. VISTA–Ig fusion protein promotes the induction of both human and murine Tregs *in vitro* (160, 165) and VISTA blockade decreases the generation of tumor-specific Tregs *in vivo* (165). This suggests that as a ligand, VISTA can promote Treg generation.

Some evidence suggests that VISTA may also be involved in the suppressive function of Tregs. First, VISTA expression is highly upregulated on tumor-infiltrating Tregs, indicating that VISTA on Tregs could play a role in suppressing tumor-specific immunity. In addition, VISTA blockade can reverse Treg-mediated suppression *in vitro*. However, VISTA blockade reverses suppression by both low VISTA expressing Tregs and high VISTA expressing Tregs and also increases the proliferation of T cells in the absence of Tregs, indicating that it might not be directly involved in Treg-mediated suppression. In this system, VISTA could function both as a receptor on T cells and a ligand expressed by Tregs (165).

**Figure 1:**
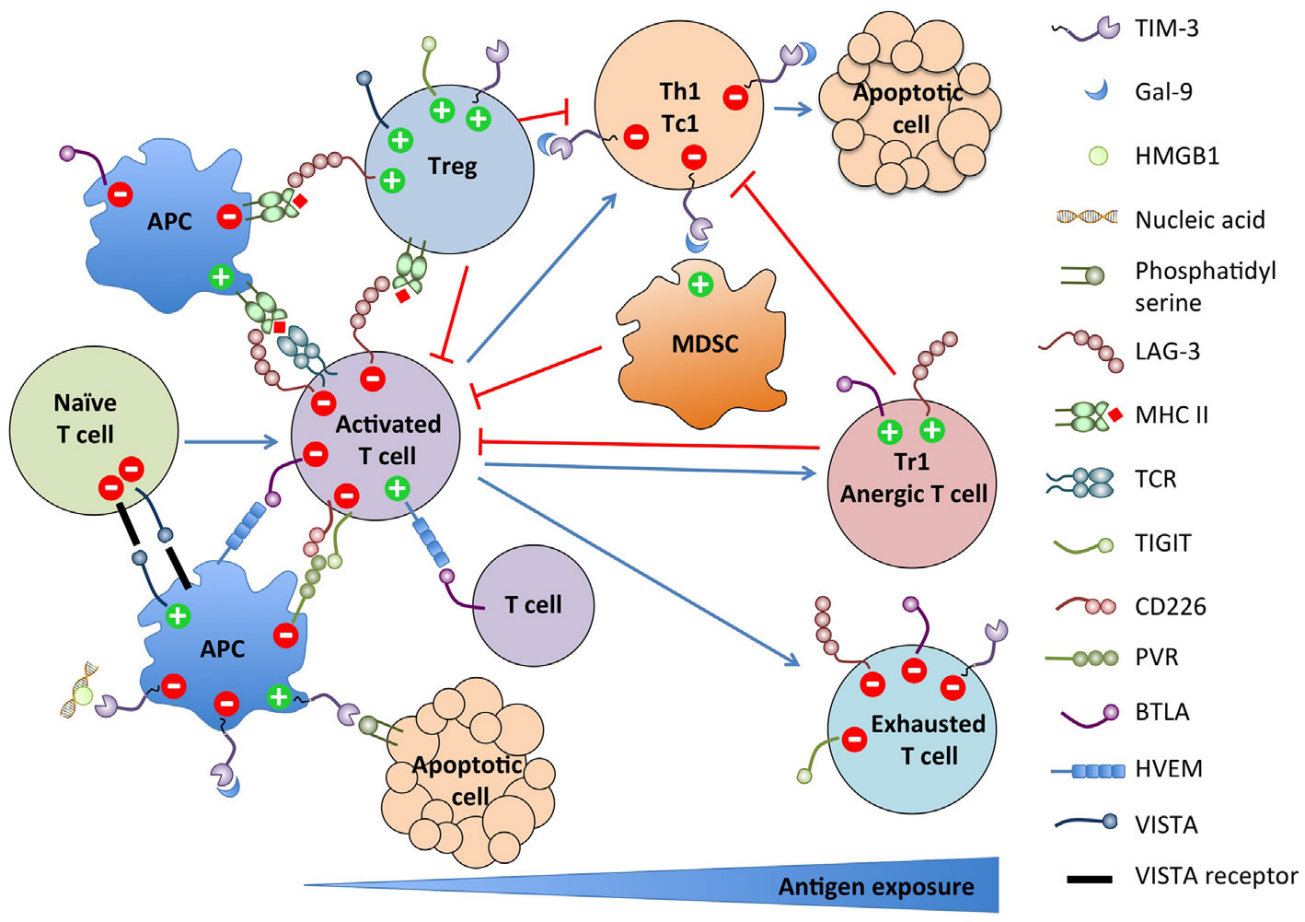
**Summary of NCRs expression on immune cell populations and their functions**.

### VISTA regulates myeloid cell activation

As mentioned, VISTA is highly expressed by myeloid cells. Its expression can be further upregulated on human monocytes by TLR ligands as well as IL-10 and IFNγ. Overexpression of VISTA is observed in monocytes from chronically HIV-infected patients. Heightened VISTA expression correlates with immune activation and CD4 depletion (166). Enforced VISTA overexpression on human monocytes/macrophages induces spontaneous secretion of multiple inflammatory cytokines at levels similar to fully activated monocytes. The process requires signaling via VISTA as cytokine secretion is abrogated by deletion of the cytoplasmic domain of VISTA. Interestingly, VISTA overexpression on HIV patients’ monocytes also enhances their ability to stimulate IFNγ production by HIV-specific T cells. Furthermore, VISTA inactivation decreases the antigen presentation ability. In this pathological situation, VISTA positive regulation of myeloid cells seems to overcome the negative signal to T cells (166). Thus, in addition to acting as a ligand for T cells, VISTA acts as a receptor on myeloid cells to regulate their activation.

### VISTA negatively regulates anti-tumor immunity

VISTA is highly expressed on tumor-infiltrating leukocytes. Importantly, it is overexpressed on MDSCs and Tregs, suggesting that VISTA plays a role in tumor evasion from the immune system (165). Indeed, VISTA-deficient mice present elevated tumor-specific immune response and are more responsive to immunization against tumor antigens (163) as well as radiotherapy treatment (161). However, VISTA deficiency alone is not sufficient to reduce tumor growth (163).

VISTA blockade also alters the suppressive character of the tumor microenvironment, reducing MDSCs, increasing DC activation and enhancing the proliferation and the effector function of tumor-infiltrating CD4 and CD8 T cells. The enhanced tumor-specific immunity results in delayed tumor growth in multiple tumor models (165). However, as most monotherapies, anti-VISTA is not sufficient to lead to complete tumor rejection. When combined with a peptide vaccine, VISTA blockade shows synergistic efficacy leading to complete tumor eradication in an inducible melanoma model (165). In addition, anti-VISTA and anti-PD-L1 combination therapy leads to tumor regression and synergistically increases tumor-specific CD8 T cell effector functions in CT26 colon carcinoma model. In non-immunogenic tumor models, combination therapy synergizes with vaccination or Treg depletion to induce tumor rejection (162).

## Concluding Remarks

As summarized in Figure 1, these new NCR pathways present striking similarities. Most NCRs are induced upon T cell activation and terminate or constrain the effector response by feedback inhibition. Some are also expressed on the APCs and regulate their stimulatory function. Conversely, multiple NCRs are expressed on Tregs and Tr1 and promote their differentiation and/or suppressive function. Most are also upregulated on dysfunctional T cells in chronic viral infections and cancer. Finally, most have multiple binding partners with which interactions are bidirectional with regard to signaling, rendering the assignment of ligand and receptor ambiguous or irrelevant. However, despite those similarities, their functions are mostly non-redundant. Therefore, blocking several of these pathways synergize in restoring efficient anti-tumor responses in preclinical models. The recently found astounding efficacy of combined anti-CTLA-4 and anti-PD-1 therapy in advanced melanoma patients argues in favor of targeting multiple pathways for future immunotherapeutic approaches (167–169).

## Conflict of Interest Statement

J. Louise Lines and Randolph J. Noelle are consultant/advisory board members for Immunext. Isabelle Le Mercier declares that the research was conducted in the absence of any commercial or financial relationships that could be construed as a potential conflict of interest.
